# Two complete mitochondrial genomes of extinct form of the Sevan trout *Salmo ischchan danilewskii*

**DOI:** 10.1080/23802359.2017.1419096

**Published:** 2017-12-21

**Authors:** Artem V. Nedoluzhko, Sergey M. Rastorguev, Evgeniy Simonov, Eugenia S. Boulygina, Fedor S. Sharko, Svetlana V. Tsygankova, Bardukh K. Gabrielyan, Haikaz R. Roubenyan, Boris A. Levin

**Affiliations:** a Genomics and Bioinformatics Laboratory, National Research Centre “Kurchatov Institute”, Moscow, Russia;; b Laboratory of Fish Ecology, Papanin Institute for Biology of Inland Waters, Russian Academy of Sciences, Borok, Yaroslavl Region, Russia;; c Laboratory of Forest Genomics,Siberian Federal University, Krasnoyarsk, Russia;; d Institute of Bioengineering, Research Center of Biotechnology of the Russian Academy of Sciences, Moscow, Russia;; e Scientific Center of Zoology and Hydroecology, National Academy of Sciences of Republic of Armenia, Yerevan, Armenia;; f Cherepovets State University, Cherepovets, Russia

**Keywords:** Mitochondrial genome, *Salmo ischchan danilewskii*, Sevan trout, illumina sequencing, historical DNA samples

## Abstract

The mitochondrial genomes from two individuals of the extinct subspecies of the Sevan trout *Salmo ischchan danilewskii* are published in this paper. The mitochondrial DNA (mtDNA) is 16,665 base pairs (bp) in length and contained 13 protein-coding genes, 2 rRNA genes, and 22 tRNA genes. The overall base composition of the genome in descending order was 27.9% of A, 29.4% of C, 16.7% of G, and 26.0% of T without a significant AT bias of 53.9%.

DNA samples from two individuals of the extinct subspecies *Salmo ischchan danilewskii*, were obtained from scales stored in an old scale book archived at the Sevan Hydrobiological Station (Sevan, Armenia). The scales were collected on 19 May 1975 in the Lake Sevan near Sarykaya (BO1 individual) (40.3603 N and 45.2379 E) and on 16 May 1974 in the Lake Sevan near vil. Tovak (BO6 individual) (40.1870 N and 45.6053 E) from fish caught by seine net.

DNA was extracted from old scales of Sevan trout in the aDNA facilities of the National Research Center ‘Kurchatov institute’ (Moscow, Russia), following the methodology described previously (Orlando et al. 2013). Two DNA libraries were prepared using an NEB Next Quick DNA Library Prep Master Mix set for 454 (New England Biolabs, Ipswich, MA) with adapter primers based on Illumina Sequencing Platform (Sarkissian et al. 2015). Mitochondrial genome was sequenced using Illumina Hiseq 1500 ((Illumina, San Diego, CA)  with 150 bp paired-end reads.

205,942,786, and 108,390,940 Illumina paired-end reads were generated for DNA library of B01 sample and B06 sample, respectively. Illumina reads from two DNA libraries were mapped to the mitochondrial genome of *S. trutta* (JQ390057) using the bowtie2 software version 2.2.3 (Langmead and Salzberg 2012) with very-sensitive-local preset options. Sequences were aligned using multiple sequence alignment program Muscle 3.8.31 (Edgar 2004). All gaps and poorly aligned positions were removed using Gblocks 0.91b (Talavera and Castresana 2007), resulting in 16,665 bp length alignment.

As a result, the mitogenome of *S. ischchan danilewskii* consists of 16,665 bp (GenBank accession numbers B01: MG599465 and B06: MG599466) and includes 13 protein-coding genes (PCGs), 2 rRNA genes, and 22 tRNA genes.

Eleven of the 13 PCGs (*NAD4, NAD5, NAD4L, NAD3, COB, NAD1, NAD2, COX2, ATP8, ATP6,* and *COX3*) used ATG as start codon, another one (*COX1*) used GTG and *NAD6* used ATA. Twelve genes (*NAD1*, *NAD2*, *COX1*, *COX2*, *ATP8*, *ATP6*, *COX3*, *NAD3*, *NAD4L*, *NAD4*, *NAD5*, and *COB*) ended with a TAA stop codon, but for three ones of them (*COX2, NAD4*, and *COB*) TAA stop codon is completed by the addition of 3′ A residues to the mRNA, *NAD6* gene ended with a TAG stop codon.

The phylogenetic analysis for whole mitogenome sequences was performed for *S. ischchan danilewskii* and other Salmonidae species: *S. trutta fario* (LC137015.1); *S. trutta* (MF621760.1); *S. trutta * (MF621762.1); *S. salar* (JQ390055.1); *S. salar* (JQ390056.1) and *Oncorhynchus kisutch* (MF621749.1) (Figure 1). The phylogenetic relationships were reconstructed using the maximum-likelihood (ML) method in the PhyML 2.4.5 (Guindon and Gascuel 2003). The best substitution model (averaged for whole mitogenome) was chosen in the jModelTest 2.1.10 (Darriba et al. 2012) on the basis of the corrected Akaike information criterion (AICc). According to jModelTest, the best model describing the evolution of the mitogenomes was GTR + G (−lnL =34,558.09, AICc =69,170.28), and therefore, it was used for ML analysis (Figure 1).

**Figure 1.**
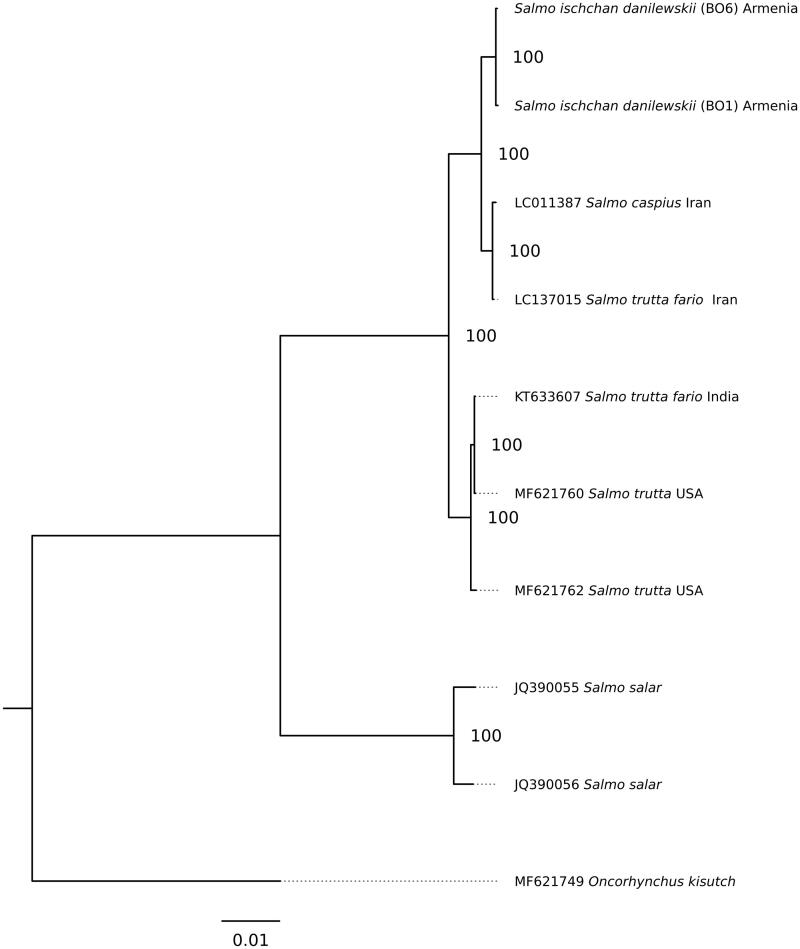
The Maximum-likelihood phylogenetic tree for *S. ischchan danilewskii* and other Salmonidae species.
